# Immune-Related Biomarkers Improve Performance of Risk Prediction Models for Survival in Patients With Hepatocellular Carcinoma

**DOI:** 10.3389/fonc.2022.925362

**Published:** 2022-07-22

**Authors:** Haifeng Wan, Shan Lu, Lin Xu, Kefei Yuan, Yang Xiao, Kunlin Xie, Hong Wu

**Affiliations:** ^1^ Department of Liver Surgery and Liver Transplantation, West China Hospital, Sichuan University, Chengdu, China; ^2^ Department of Breast Surgery, West China Hospital, Sichuan University, Chengdu, China; ^3^ Laboratory of Liver Surgery, West China Hospital, Sichuan University, Chengdu, China; ^4^ Department of Plastic and Burn Surgery, West China Hospital, Sichuan University, Chengdu, China

**Keywords:** hepatocellular carcinoma, immune-related biomarkers, survival, prediction, prognostic model

## Abstract

**Object:**

The prediction of hepatocellular carcinoma (HCC) prognosis faced great challenge due to tumor heterogeneity. The purpose of this study was to explore the correlation between the immune infiltrate and prognosis. Moreover, we aimed to establish a risk prediction model for survival in HCC patients based on clinicopathological and immune indicators.

**Methods:**

In this study, 316 patients with HCC who underwent radical resection in West China Hospital from 2009 to 2014 were included. Clinicopathological data and pathological specimens were collected. H&E staining and immunohistochemical staining were performed on the pathological tissue sections. The evaluation of tumor-infiltrating lymphocyte (TIL) density was based on H&E slices, and the assessment of the expressions of CD8, CD68, Lymphocyte activation gene-3 (LAG-3), T cell immunoglobulin domain and mucin domain-3 (TIM-3), Programmed Cell Death Protein 1 (PD-1), Programmed Cell Death Ligand 1 (PD-L1), OX40, CD66b, and Tryptase. was performed on the immunohistochemical slices. A risk prediction model for survival in HCC patients was established by integrating immune-related biomarkers and clinicopathological indicators.

**Results:**

The Barcelona Clinic Liver Cancer (BCLC) stage; the microvascular invasion status; the density of TILs; the expressing levels of CD66b, OX40, and PD-L1 in the immune cell; CD68; and CD8 were the predictors of patients’ overall survival (OS). The BCLC stage; the density of TILs; and the expressions of OX40, CD68, and CD8 were associated with disease-free survival (DFS). The expressions of CD66b, CD68, OX40, and CD8 had a cumulative effect on prognosis. The area under the curve of the prediction model for OS based on clinicopathological features was improved from 0.62 to 0.74 by adding to CD8, OX40, CD68, CD66b, and TILs, whereas it was improved from 0.59 to 0.73 for the DFS prediction model.

**Conclusion:**

Our results, if confirmed, indicated that immune-related biomarkers should be taken into account or stratified in survival analysis for HCC.

## Introduction

Hepatocellular carcinoma (HCC) is the sixth most prevalent malignant tumor and the fourth leading cause of cancer-related death worldwide (1). Although a comprehensive strategy consisting of surgical resection, liver transplantation, ablation, transarterial chemoembolization (TACE), and targeted therapy applied to HCC patients has shown its therapeutic efficacy in curbing overall mortality from this disease, the recurrence and mortality remain an escalating trend in many countries, and the advanced stage is the predominant state of HCC patients in many parts of the world (2). More attention should be paid to patients at high risk of recurrence in order to improve patients’ overall survival (OS) (3, 4). An integrated prognostic predicting system for HCC patients who underwent radical resection may facilitate patient stratification and individualized follow-up planning.

In the past decades, several prognostic models based on clinicopathological data such as the Japan Integrated Staging score (5), the Cancer of the Liver Italian Program score (6), and the Barcelona Clinic Liver Cancer (BCLC) staging system (7) have been proposed for HCC and widely accepted. However, there is no consensus regarding the optimal tool for prognosis stratification.

The tumor immune microenvironment, determined by the density, composition, functional state, and organization of the leukocyte infiltrate of the tumor, is a focus in the studies of tumor immunology (8, 9). The commonly investigated subsets of leukocytes in the tumor immune microenvironment include T cells, B cells, natural killer cells, tumor-associated macrophages (TAMs), neutrophil leukocytes, and even mast cells. Accumulating evidence suggests that different leukocyte subsets have different impacts on tumorigenesis and immune response, indicating different outcomes. For example, CD8+ T cells constitute the most powerful immune attack against tumors and higher tumor infiltration of CD8+ T cells showed a significant advantage in OS or DFS (10–12), whereas it is reported that T regulatory cells (Tregs) were associated with worse survival in gastrointestinal tumors (13–15). An elevated neutrophil ratio showed poor survival (16, 17), and high infiltration of TAMs was correlated with tumor recurrence and shorter survival due to the possible suppression of effective anticancer immunity, the stimulation of angiogenesis, and tissue remodeling (18, 19). Mast cells are another subset of immune cells, which were reported to accumulate in both solid and hematologic tumors. The consequences of their presence in the tumor immune microenvironment still remain unclear (20–22). In recent years, immune checkpoint therapy has been widely used to treat malignant cancers (23–25). Immune checkpoints such as Programmed Cell Death Protein 1 (PD-1), T cell immunoglobulin domain and mucin domain-3 (TIM-3), and Lymphocyte activation gene-3 (LAG-3), which are often activated in the tumor tissue and regulate T-cell function, are important immunotherapy targets (26–28). PD-1 inhibitors have shown promising clinical effects in the treatment for HCC (29). The tumor immune microenvironment has shown significant values (30, 31). However, additional evidence and further exploration are still needed.

In the present study, we aimed to identify the immune-related predictors for HCC patients after curative resection and analyzed their effects on OS and disease-free survival (DFS). Furthermore, we tried to establish a novel risk prediction model for survival in HCC patients based on clinicopathologic and immune variables, aiming at improving the management of high-risk groups.

## Materials and methods

### Patients and Samples

The research protocol was approved by the local ethics committee of West China Hospital (2019788), and written informed consent was provided in this study. From 2009 and 2014, consecutive patients who fulfilled the following inclusion criteria were enrolled: a) age ≥ 18 years and ≤70 years and b) had surgically confirmed HCC and underwent radical resection. Patients were excluded if they: a) had any preoperative anticancer treatments for HCC; b) had any postoperative systemic anticancer treatments for HCC; c) had a history of other malignant tumors; and d) died within 2 weeks postoperatively due to any complication.

The sample collection was as previously described (32). Briefly, approximately 5-g surgical specimens of tumor and adjacent tissue were isolated once resected and immediately stored in liquid nitrogen until analysis. Tissue cores were punched from the representative tissue areas of HCC samples that were fixed by formalin and embedded by paraffin orderly and arranged in 1 recipient paraffin block, which was cut into 5-mm sections.

### Data Collection

Clinical and biological data, including age, gender, hepatitis B and C status, and the serum alpha-fetoprotein (AFP) level were obtained before operation from an electronic clinical database for analysis. The Barcelona Clinic Liver Cancer (BCLC) staging system was used to evaluate tumor staging at the time of each patient’s diagnosis. Histological slides were reviewed for the tumor size, number, differentiation, vascular invasion, and the Ishak fibrosis score of the adjacent liver tissue by a pathologist specializing in hepatobiliary pathology and tumor characteristics.

### Follow-up

All patients were followed up after surgery at 1 month, every 3–6 months thereafter with serum AFP and ultrasound, contrast-enhanced CT, and/or contrast-enhanced MRI. The primary endpoint was OS, defined as the time from surgery to death regardless of the cause. The secondary end point was DFS, defined as the interval between surgery and the detection of recurrence or death from any cause. Patients with no events were censored at the time of their last follow-up.

### Hematoxylin and Eosin Stain and Immunohistochemistry

H&E stain and immunohistochemistry were performed according to standard protocols detailed in previous studies (32, 33). The primary antibodies used for immunohistochemistry (IHC) contained the T-cell marker of CD8; the macrophage marker of CD68; the Treg marker of OX40; the neutrophil marker of CD66b; the mast cell marker of Tryptase; and the immune checkpoints of PD-1, PD-L1, TIM-3, and LAG3. The details are shown in **Table S1**. The NDP.view.2 software program was used to view and capture the images for analysis.

### Pathologic Evaluation

**Figure 1:**
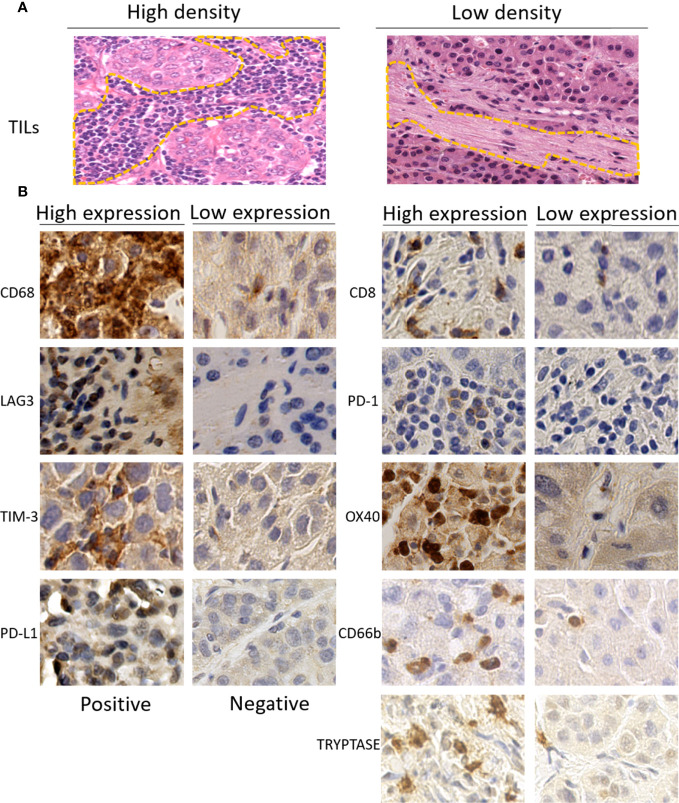
**(A)** Representative images of high (left) or low (right) density of tumor-infiltrating lymphocytes (TILs) in the stromal of hepatocellular carcinoma on H&E-stained tumor sections (×100). **(B)** Representative immunohistochemistry images of high (left) or low (right) expression of immune-related biomarkers (×400). TILs, tumor-infiltrating lymphocytes.

Pathologic evaluation was conducted by two liver pathologists blinded to clinical data. The quantitative assessment of TILs in tumor stromal was carried out on H&E-stained slides with 100 times magnification according to International TILs Working Group criteria (34). The full section was scanned for assessment except in areas with crush artifacts, necrosis, and regressive hyalinization. A full assessment of average TILs in the tumor area was used. Additionally, the scores were further classified into low (TILs < 10%)- and high (TILs ≧ 10%)-TIL groups (**Figure 1A**).

As described in a previous study (32), five random images at 400 times magnification for each immune-stained slice were used for IHC evaluation. In brief, the bleeding areas, necrotic areas, marginal areas of non-specific staining, and non-tumor cell areas were excluded firstly. The tumor areas were then divided into a number for regions and numbered with Arabic numbers on scanning slice. Five random numbers were generated using Excel software to select five corresponding areas for quantitative assessment. The measurements for CD8, CD68, PD-1, TIM-3, LAG3, OX40, CD66b, and Tryptase were recorded as the total number of positive-expressing cells. The expressions of these immune-related markers were categorized into the high group, defined as the number of positive immune cells superior to the median of the full series, and the low group, defined as the number equal or inferior to the median of the full series. The counts of the expression of PD-L1 were performed in tumor-infiltrating immune cells and tumor cells, respectively. The classification for the PD-L1 expressed in tumor-infiltrating immune cells was the same as the classification for the other markers described above, while the expressions of PD-L1 in tumor cells were grouped as positive if the number of positive cells reached 1% tumor cell staining on the membrane. The representative immunohistochemical staining images of immunomarkers are shown in **Figure 1B**.

### Statistical Analysis

All statistical tests were two sided, and P-values less than 0.05 were considered to be statistically significant. Differences between groups were compared by Student’s t-test for continuous variables and by the χ^2^ test or Fisher’s exact test for categorical variables. The survival analysis was estimated using the Kaplan–Meier method, and OS and DFS were assessed using the log-rank test. The Cox proportional hazards regression method was used to identify the risk predictors of HCC. The hazard ratios (HRs) and 95% CI were estimated for immune-related biomarkers (low/high or positive/negative) in both univariate and multivariate analysis models and tumor stage (BCLC A/B/C) and the microvascular invasion (MVI) status (yes/no) in the multivariate analysis model. The immune-related biomarkers with P less than 0.20 in the univariate were used for multivariate analysis. Cases with missing data items were also included in the analysis by categorizing them as “missing.” The receiver operating characteristic (ROC) curve is used to detect the effectiveness of the risk prediction model, and Kruskal–Wallis is used to analyze and compare the area under the ROC curve. Statistical analysis was performed using R software (R 3.3.2). Reporting Recommendations for Tumor Marker Prognostic Studies (REMARK) criteria were followed for this study (35).

## Results

### Characteristics of Study Populations

A total of 316 patients fulfilling the inclusion and exclusion criteria were enrolled in this study. The baseline characteristics of the patients are summarized in **Table S2**. All patients were from China, and there were 258 men, accounting for 81%. The median follow-up time was 34 months (range 1–93 months).

### Prognostic Value of Immune-Related Features

The results of the univariate logistic regression analysis of the immune-related biomarkers are shown in **Table S3**. TILs density, expressions of CD66b, OX40, CD68 and CD8 were associated with OS. TILs density and expression of OX40 were associated with DFS. Additionally, all these factors with a P-value less than 0.2 were selected for the multivariate analysis with the BCLC stage and MVI status, which were generally accepted as prognostic factors for HCC. In the multivariate analysis, the BCLC C stage; the MVI status; the density of TILs; and the expressions of CD66b, OX40, and PD-L1 in tumor-infiltrating immune cells, CD68, and CD8, were significantly associated with OS. Meanwhile, the BCLC stage; TIL density; and the expressions of OX40, CD68, and CD8 were significant risk predictors for DFS (**Table 1**).

**Table 1:** Prognostic value of clinicopathological and immune-related parameters for overall survival (OS) and disease-free survival (DFS) estimated from the multivariable model.

(n = 316)									
Variables	OS				DFS			
	Patients, No. (%)	Incidence, No. (%)	HR, (95% CI)	P	Patients, No. (%)	Incidence, No. (%)	HR, (95% CI)	P
BCLC Stage								
A B C	218(78.4) 47(16.9) 13(4.7)	61(28.0) 19(40.4) 10(76.9)	1.00 1.63 (0.92–2.87) 3.30 (1.56–6.99)	0.09 <0.01	121(73.3) 31(18.8) 13(7.9)	63(52.1) 21(67.7) 12(92.3)	1.00 1.75(1.04–2.95) 2.30(1.16–4.55)	0.04 0.02
MVI								
No Yes	219(69.3) 97(30.7)	56(25.6) 43(44.3)	1.00 1.58 (1.00–2.49)	0.05	219(69.3) 97(30.7)	63(28.8) 43(44.3)	1.00 1.28(0.84–1.96)	0.25
TILs								
Low High	175(55.6) 140(44.4)	70(40.0) 29(20.7)	1.00 0.39 (0.25–0.63)	<0.01	103(56.3) 80(43.7)	80(77.7) 26(32.5)	1.00 0.29 (0.18–0.46)	<0.01
CD66b								
Low High	149(50.0) 149(50.0)	30(20.1) 62(41.6)	1.00 1.89 (1.19–3.00)	<0.01				
OX40								
Low High	180(57.0) 136(43.0)	39(21.7) 60(44.1)	1.00 3.52 (2.20–5.60)	<0.01	99(53.8) 85(46.2)	50(50.5) 56(65.9)	1.00 2.80 (1.74–4.50)	<0.01
PD-L1_immune								
Negative Positive	236(81.1) 55(18.9)	71(30.1) 23(41.8)	1.00 2.13 (1.25–3.63)	<0.01				
PD-1								
Low High	272(86.3) 43(13.7)	89(32.7) 10(23.3)	1.00 0.62 (0.29–1.31)	0.21				
LAG3								
Low High	178(57.0) 134(43.0)	48(27.0) 51(38.1)	1.00 1.20 (0.76–1.88)	0.44	95(52.5) 86(47.5)	52(54.7) 54(62.8)	1.00 1.07 (0.69–1.67)	0.75
CD68								
Low High	159(50.8) 154(49.2)	37(23.3) 62(40.3)	1.00 1.75 (1.10–2.78)	0.02	85(46.7) 97(53.3)	43(50.6) 63(64.9)	1.00 1.74 (1.12–2.68)	0.01
CD8								
Low High	157(50.3) 155(49.7)	65(41.4) 33(21.3)	1.00 0.28 (0.17–0.44)	<0.01	77(42.5) 104(57.5)	48(62.3) 58(55.8)	1.00 0.51 (0.33–0.80)	<0.01

No, number; HR, hazard ratio; CI, confidence interval; OS, overall survival; DFS, disease-free survival; BCLC Stage, Barcelona Clinic Liver Cancer Stage; MVI, microvascular invasion; TILs, tumor-infiltrating lymphocytes.

### Cumulative Effects of Immune-Related Markers on Prognosis

**Figure 2:**
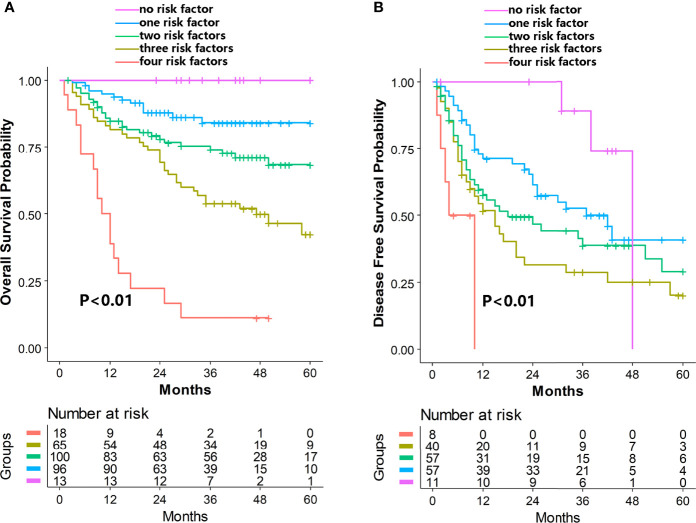
Cumulative effects of immune-related markers on OS **(A)** and DFS **(B)** in hepatocellular carcinoma. High expression of CD66b, CD68, or OX40 and low expression of CD8 were identified as the independent risk factors. The purple, blue, green, yellow, and red lines represent the group of patients with none, one, two, three, and four risks, respectively. OS, overall survival; DFS, disease-free survival.

Based on the multivariate analysis results that high expression of CD66b, CD68, or OX40 and low expression of CD8 were risk factors for both OS and DFS, the participants in this study were further categorized into five groups by the number of these risk factors. The survival curves showed that the group with no risk factor had the best OS, which declined with an increasing number of risk factors. A similar result was observed for DFS (**Figure 2**).

### CD8:OX40 Ratio was Associated With Hepatocellular Carcinoma Prognosis and Clinicopathologic Features

**Figure 3:**
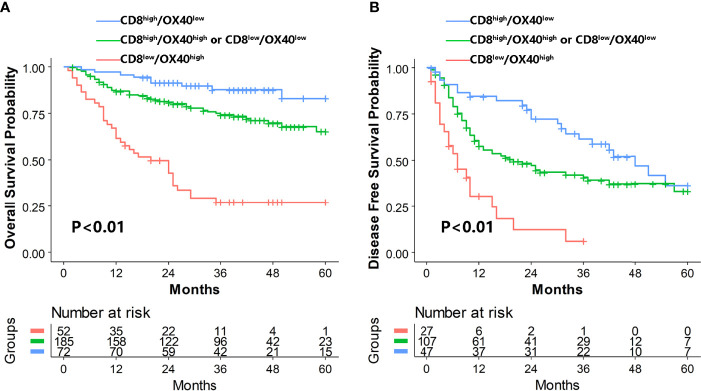
Kaplan–Meier analysis of OS **(A)** and DFS **(B)** for CD8+/OX40+ TIL ratio. OS, overall survival; DFS, disease-free survival; TILs, tumor-infiltrating lymphocytes.

The prognostic values of CD8 and OX40 in our study were in accordance with previous studies (32, 36, 37).The prognostic relevance of the ratio of CD8/OX40 was investigated. We observed that the high-ratio group (CD8^high^/OX40^low^) and intermediate-ratio group (CD8^high^/OX40^high^ or CD8^low^/OX40^low^) had better OS (high-ratio group HR=0.10,95%CI=0.04–0.21, intermediate-ratio group HR=0.25,95%CI=0.16–0.39, P *_for trend_* <0.01) and better DFS (high-ratio group HR=0.21,95%CI=0.11-0.39, intermediate-ratio group HR=0.33,95%CI=0.20–0.56, P *_for trend_* <0.01) compared to the low-ratio group (CD8^low^/OX40^high^) (**Table 2**, **Figure 3**). Further analysis indicated that the high-ratio group was associated with a higher AFP level (>400 ng/ml), an MVI-positive status, multiple tumors, and a smaller tumor diameter (median = 4 cm) in comparison to the low-ratio group (**Table 3**).

**Table 2:** Univariate analysis of the ratio of CD8 and OX40 expressions for OS and DFS.

Variables	OS				DFS			
	No. of alive	No. of death	HR (95% CI)	P	No. of recurrence-free	No. of recurrence	HR (95% CI)	P
CD8^low^/OX40^high^	16	36	1(reference)		6	21	1(reference)	
CD8^high^/OX40^high^ or CD8^low^/OX40^low^	135	52	0.25(0.16-0.39)	<0.01	45	62	0.33(0.20–0.56)	<0.01
CD8^high^/OX40^low^	63	10	0.10(0.04-0.21)	<0.01	24	23	0.21(0.11–0.39)	<0.01
*P*_for trend_				<0.01				<0.01

No., number; HR, hazard ratio; CI, confidence interval; OS, overall survival; DFS, disease-free survival.

**Table 3:** Association between the CD8+/OX40+ ratio and clinicopathological parameters.

	CD8^low^/OX40^high^	CD8^high^/OX40^high^ or CD8^low^/OX40^low^	CD8^high^/OX40^low^	P
AFP>400(ng/ml)	25/51(49%)	117/187(62.6%)	56/73(23.3%)	<0.01
MVI	26/52(50%)	58/187(31%)	12/73(16.4%)	<0.01
No. of tumor>1	15/48(31.3%)	25/161(15.5%)	10/62(16.1%)	0.04

Data are no. of patients (%) unless otherwise indicated.

No., number; AFP, alpha-fetoprotein; MVI, microvascular invasion.

### Prognostic Model Establishment and Assessment

**Figure 4:**
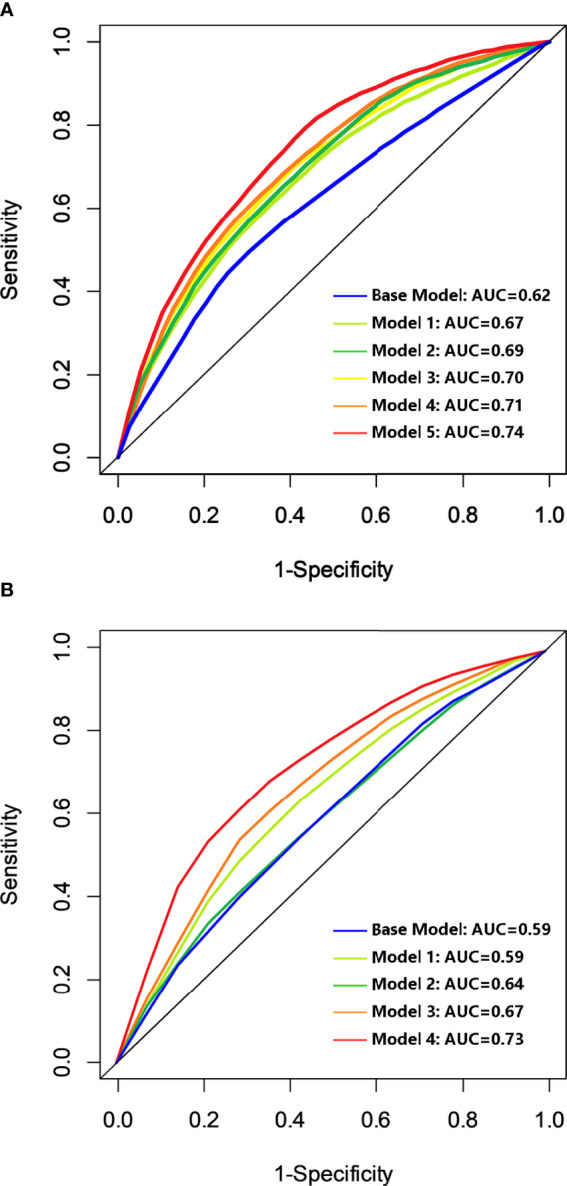
Discriminatory accuracy of the models for OS **(A)** and DFS **(B)**. Base model was based on the BCLC stage, differentiation, MVI status, and cirrhosis status; model 2 to model 5 were built by adding predictive immunologic variables of CD8, OX40, CD68, CD66b, and TILs successively for OS. Model 2 to model 4 were built by adding the predictive immunologic variables of CD8, OX40, CD66b, and TILs successively to the base model for DFS. OS, overall survival; DFS, disease-free survival; AUC, area under the curve; BCLC stage, Barcelona Clinic Liver Cancer stage; MVI, microvascular invasion; TILs, tumor-infiltrating lymphocytes.

The base model of risk prediction for OS was established using the well-known clinicopathologic prognostic variables including the BCLC stage, tumor differentiation, MVI status, and cirrhosis status, with an area under the curve (AUC) of 0.62. Five novel predictions were built by adding predictive immune-related biomarkers successively selected from the aforementioned analysis, containing CD8, OX40, CD68, CD66b, and TILs to the base model. Model 2 was based on the base model and immune-related biomarker of CD8; Model 3 was based on the base model and immune-related biomarkers of CD8 and OX40 together. Like this, model 5 was constructed based on the base model and CD8, OX40, CD68, CD66b, and TILs. The AUC values were 0.67, 0.69, 0.70, 0.71, and 0.74, respectively (**Figure 4A**). The model 5 inputs including 4 clinicopathologic variables and 5 immune-related biomarkers for OS resulted in the highest AUC value of 0.74. The base model for DFS was also established based on the BCLC stage, tumor differentiation, MVI status, and cirrhosis status, with an AUC of 0.59. Meanwhile, four novel prediction models were developed by adding the immune-related biomarkers of CD8, OX40, CD66b, and TILs to the base model one by one. The AUC values were 0.59, 0.64, 0.67, and 0.73 respectively (**Figure 4B**). The model 4 inputs containing both 4 clinicopathologic variables and 4 immune-related biomarkers for DFS turned out the highest AUC value of 0.73.

## Discussion

In this study, we examined the relationship between the individual immune subset of the infiltrates in the HCC microenvironment and patients’ mortality. As is known, the CD8+T cell, serving as the main effector cell, played a crucial role in antitumor immunity (38). Conversely, OX40+ Tregs exerted a strong immunosuppressive function, especially in collaboration with other immunosuppressive cells, such as macrophages and dendritic cells, leading to the depletion of T cells (39–41). Our results observed positive and negative associations of CD8+T cell and OX40+ Tregs, respectively, with OS and DFS. Moreover, we tested whether the ratio of CD8 and OX40 was associated with the clinicopathologic parameters. We did find that the ratio of CD8:OX40 was relevant to the AFP level, MVI-positive status, tumor number, and tumor diameter, which were identified as the risk factors for survival in another study (42).

The intensity of TILs serving as a biomarker regardless of the intricate heterogeneity of subsets was also an attempt for prognostic assessment. The TIL assessment was kept to the criteria proposed by the International TILs Working Group. In line with other solid tumors such as esophageal cancer and melanoma (43, 44), TIL intensity was proven effective for prognosis prediction for HCC. The classification of the percentage of TILs in our study was slightly different from the method proposed by the International TILs Working Group. As the number of patients with TIL intensity above 50% was too small, we combined those with TIL intensity between 10% and 50% and those higher than 50%, which were categorized as intermediate- and high-TIL groups, respectively, by the International TILs Working Group, into one group. The result that higher TIL density was in favor of survival was consistent with the results in breast cancer and non-small cell lung cancer studies (45, 46). A possible explanation could be that TIL infiltration was more likely to occur in tumors that are more immunogenic (47, 48).

We identified a prominent accumulative negative effect on prognosis as the number of risk factors increased. A possible explanation could be the synergistic effects of different subsets of infiltrate. CD68 and CD66b were generally expressed on the TAMs and neutrophils, respectively. There was plenty of evidence suggesting that both of them could promote neovascularization and were associated with tumor invasion and metastasis (49–52). The depletion of T cells caused by these immunosuppressive cells could also be a reason for the associated poor prognosis (39–41). The role of tryptase, a marker of mast cells, in tumors remains controversial. Some studies have shown that mast cells infiltrated in the tumor microenvironment were associated with a poor prognosis (53–55). Others have indicated that mast cells contain a variety of pro-inflammatory mediators. Additionally, the unique ability to prestore and release potentially beneficial anticancer mediators such as tumor necrosis factor α (TNF-α) and the granulocyte–macrophage colony-stimulating factor, mediating the necrosis of cancer cells (56–58), is prognostic. However, the fact that a prognostic value of tryptase was not observed in our study may be related to limited cases and missing immunohistochemical data in some cases. In addition, PD-1, TIM-3, and LAG3 did not have independent prognostic significance in our study, which may not limit their potential roles as predictive biomarkers for prognosis.

The concept of immune contexture, representing the preexisting immune parameters associated with patient survival, was generally accepted. The immune contexture can be leveraged not for therapeutics but rather for classification purposes (31), whereas the traditional alternative cancer classification modalities are still in use due to the poor exploitation of the immune contexture. In this study, we investigated the prognostic value of the immune features, identified the significant immunologic risk factors, and developed a prediction model for HCC based on both clinicopathologic and immune cell infiltrate subsets. We did find that the prognostic predictions integrating clinicopathologic and immune-related variables could improve the discrimination. This model is a tool for prognosis risk stratification and can be used to determine survival prognosis, which can assist clinicians in drawing up comprehensive treatment plans for patients and provide scientific assistance for surgical prognosis preliminary judgment and a basis for adjuvant therapy. It has clinical significance for prognosis prediction and a personalized management of HCC patients.

There are several limitations to our study. First, although our models demonstrated a relatively excellent discriminatory ability, external confirmation is needed. The similar results in one or more different cohorts, especially with a large population, are able to enhance the reliability of our finding. Second, our study did not document the prognostic meaning of some other immune-related markers. However, we have investigated those reported to play a significant role in HCC. Furthermore, the retrospective design of this study is another limitation.

In summary, integrating immune-related biomarkers based on the clinicopathologic model can improve the risk-predictive performance for survival in HCC patients, indicating that immune-related biomarkers should be taken into account or stratified in survival analysis for HCC.

## Data Availability Statement

The raw data supporting the conclusions of this article will be available from the corresponding author upon reasonable request.

## Ethics Statement

The studies involving human participants were reviewed and approved by ethics committee of West China Hospital. The patients/participants provided their written informed consent to participate in this study.

## Author Contributions

HW, HFW, and KX designed the experiments. HFW, KX, and LX performed the experiments. HFW and SL collected the clinical data. HFW, KX, and SL analyzed the data and made the figures. All authors wrote and revised the manuscript. HW proposed the study concept, supervised the research, and acquired funding for the study. All authors reviewed the manuscript. All authors contributed to the article and approved the submitted version.

## Funding

This work was supported by grants from the National Key Technologies R&D Program (2018YFC1106800), the National multidisciplinary collaborative diagnosis and treatment capacity building project for major diseases (TJZ202104), the Natural Science Foundation of China (82173124, 82173248, 82103533, 82002572, 82002967, 81972747, and 81872004), the fellowship of China National Postdoctoral Program for Innovative Talents (BX20200225, BX20200227), the China Postdoctoral Science Foundation (2021M692278, 2020M673231), the Science and Technology Support Program of Sichuan Province (2021YJ0436), the Postdoctoral Science Foundation of Sichuan University (2021SCU12007), the 1.3.5 project for disciplines of excellence, West China Hospital, Sichuan University (ZYJC18008), and the Postdoctoral Science Foundation of West China Hospital (2020HXBH075, 2020HXBH007).

## Conflict of Interest

The authors declare that the research was conducted in the absence of any commercial or financial relationships that could be construed as a potential conflict of interest.

## Publisher’s Note

All claims expressed in this article are solely those of the authors and do not necessarily represent those of their affiliated organizations, or those of the publisher, the editors and the reviewers. Any product that may be evaluated in this article, or claim that may be made by its manufacturer, is not guaranteed or endorsed by the publisher.
