# Supplementary material for: Immune-Related Biomarkers Improve Performance of Risk Prediction Models for Survival in Patients With Hepatocellular Carcinoma
Source: Front Oncol. 2022 Jul 22;12:925362. doi: 10.3389/fonc.2022.925362 (PMC9353009; doi:10.3389/fonc.2022.925362)
Supplement: Supplementary file 1 [file Table_1.docx]

**Supplementary information**

| Table S1. Primary antibodies used in the study | | | | |
| --- | --- | --- | --- | --- |
| **Antigens** | **Source** | **Clone** | **Retrieval buffer** | **Dilution** |
| CD66b | Abcam | ab197678 | Citrate buffer, PH = 6 | 1 : 100 |
| TRYPTASE | Abcam | Ab2378 | Citrate buffer, PH = 6 | 1 : 100 |
| OX40 | Abcam | ab119904 | Citrate buffer, PH = 6 | 1 : 100 |
| PD-1 | Abcam | ab52587 | Citrate buffer, PH = 6 | 1 : 50 |
| PD-L1 | CST | #13684 | Citrate buffer, PH = 9 | 1 : 200 |
| TIM-3 | R&D Systems | AF2365-SP | Citrate buffer, PH = 6 | 2 µg/ml |
| LAG3 | LifeSpan Bioscience | LS-B2237 | Citrate buffer, PH = 6 | 1 : 100 |
| CD68 | DAKO | M087601-2 | Citrate buffer, PH = 6 | 1 : 100 |
| CD8 | Thermo Scientific | MA5-1347 | Citrate buffer, PH = 6 | 1 : 50 |

Table S2. Characteristics of patients in the West China Hospital

| Characteristic | (n=316) |
| --- | --- |
| Sex |  |
| Men | 258 (81) |
| Women | 58 (19) |
| Age, years |  |
| Mean | 51.28 |
| ≤40 | 55 (19) |
| 40-60 | 181 (54) |
| ≥60 | 80 (27) |
| Race |  |
| Asian | 316 (100) |
| White | 0 |
| Others | 0 |
| Risk factor |  |
| HBV | 214 (99) |
| HCV | 3 (1) |
| Alcohol | 0 |
| Nonalcoholic steatohepatitis | 0 |
| Other | 0 |
| No history of primary risk factors | 0 |
| NA | 99 |
| Serum AFP level, ng/ml |  |
| >20 | 181 (58) |
| ≤20 | 131 (42) |
| No. of tumors |  |
| 1 | 227 (82) |
| >1 | 51 (18) |
| Mean tumor size (range), cm | 5.98 (0.3-19) |
| BCLC stage |  |
| A | 218 (78) |
| B | 47 (17) |
| C | 13 (5) |
| Vascular invasion |  |
| Microvascular | 97 (31) |
| None | 219 (69) |
| NA |  |
| Tumor differentiation |  |
| I | 7 (2) |
| II | 160 (55) |
| III/IV | 122 (42) |
| NA | 27 |
| Ishak score |  |
| 0 | 70 (25) |
| 1-2 | 10 (4) |
| 3-4 | 59 (21) |
| 5-6 | 142 (50) |
| NA | 35 |
| No. of deaths | 99 (31) |
| Median follow-up time (range), months | 34 (1-93) |

| **Note:** Data are no. of patients (%) unless otherwise indicated.  **Abbreviations:** HBV, hepatitis B virus; HCV, hepatitis C virus; AFP, alpha fetoprotein; BCLC, Barcelona Clinic Liver Cancer; AJCC, American Joint Committee on Cancer; NA, not available. |
| --- |

Table S3. Prognostic value of immune-related markers for OS and DFS estimated from univariable model.

|  | | | | | | |  |
| --- | --- | --- | --- | --- | --- | --- | --- |
| (n = 316) | | | | | | |  |
|  | OS | |  |  | DFS | |  |
| Variable | Events/No.  of Patients | HR (95% CI) | P |  | Events/No.  of Patients | HR (95% CI) | P |
| TILs  Low  High | 70/175  29/140 | 1.00  0.49 (0.32-0.75) | <0.01 |  | 80/103  26/80 | 1.00  0.30 (0.20-0.48) | <0.01 |
| CD66b  Low  High | 30/149  62/149 | 1.00  1.73 (1.11-2.69) | 0.01 |  | 52/97  48/80 | 1.00  1.14 (0.77-1.70) | 0.49 |
| TRYPTASE  Low  High | 27/87  67/224 | 1.00  1.21 (0.77-1.91) | 0.39 |  | 25/46  79/136 | 1.00  1.61 (0.73-1.82) | 0.52 |
| OX40  Low  High | 39/180  60/136 | 1.00  2.77 (1.83-4.18) | <0.01 |  | 50/99  56/85 | 1.00  2.12 (1.44-3.13) | <0.01 |
| PD-L1_tumor  Negative  Positive | 90/258  10/33 | 1.00  0.88 (0.47-1.66) | 0.70 |  | 90/258  10/33 | 1.00  0.78 (0.40-1.52) | 0.46 |
| PD-L1_immune  Negative  Positive | 71/236  23/55 | 1.00  1.58(0.98-2.53) | 0.06 |  | 78/236  22/55 | 1.00  1.29(0.80-2.07) | 0.30 |
| PD-1  Low  High | 89/272  10/43 | 1.00  0.51 (0.24-1.05) | 0.13 |  | 87/152  19/31 | 1.00  0.90(0.54-1.47) | 0.67 |
| TIM-3  Low  High | 48/155  50/154 | 1.00  1.06 (0.72-1.56) | 0.77 |  | 46/80  58/99 | 1.00  1.06 (0.72-1.56) | 0.78 |
| LAG3  Low  High | 48/178  51/134 | 1.00  1.48 (1.00-2.21) | 0.05 |  | 52/95  54/86 | 1.00  1.37 (0.94-2.01) | 0.11 |
| CD68  Low  High | 37/159  62/154 | 1.00  2.03 (1.34-3.07) | <0.01 |  | 43/85  63/97 | 1.00  1.47 (1.00-2.17) | 0.05 |
| CD8  Low  High | 65/157  33/155 | 1.00  0.44 (0.29-0.68) | <0.01 |  | 48/77  58/104 | 1.00  0.73 (0.50-1.07) | 0.11 |

**Abbreviation:** No, number; HR, hazard ratio; CI, confidence interval; OS, overall survival; DFS, disease-free survival; TILs, tumor-infiltrating lymphocytes.
